# Do Chinese Traditional and Modern Cultures Affect Young Adults’ Moral Priorities?

**DOI:** 10.3389/fpsyg.2018.01799

**Published:** 2018-11-06

**Authors:** Xiaomeng Hu, Sylvia Xiaohua Chen, Li Zhang, Feng Yu, Kaiping Peng, Li Liu

**Affiliations:** ^1^Department of Psychology, Tsinghua University, Beijing, China; ^2^Department of Applied Social Sciences, The Hong Kong Polytechnic University, Hung Hom, Hong Kong; ^3^Institute of Social Psychology, Xi’an Jiaotong University, Xi’an, China; ^4^Beijing Key Lab of Applied Experimental Psychology, School of Psychology, Beijing Normal University, Beijing, China

**Keywords:** Chinese traditional culture, Chinese modern culture, moral priorities, cultural priming, young adults

## Abstract

Dramatic cultural change has occurred in Mainland China over the past four decades, yet little is known about how this cultural shift impacts Chinese peoples’ moral values. The present research aims to fill this gap by examining whether Chinese traditional and modern cultures influence young adults’ moral judgments. Study 1 investigated the relation between psychological traditionality/modernity and moral concerns. Results indicated that participants who strongly endorsed Chinese traditional culture prioritize relationship concern rather than justice concern. Study 2 used the cultural priming method and tested the effects of traditional and modern icons on moral concerns. Results suggested that participants who were primed with traditional or modern or neutral icons did not give priority to relationship or justice concern. Together, our findings provide initial empirical evidence on whether Chinese traditional and modern cultures shift the moral mindsets of bicultural young Chinese among alternative (and even competing) moral codes.

## Introduction

The rapid and deep societal change in Mainland China over the past four decades has profoundly reshaped the everyday life of a billion people. A variety of cultural theories have been proposed to describe and explain how and why cultures change at the societal level (Inkeles and Smith, 1974; Huntington, 1997; Inglehart and Baker, 2000). At the individual level, psychologists have formulated informative theories and testable hypotheses to examine whether individual differences in traditionality and modernity map onto a set of psychological processes and behavioral patterns (Yang, 1996, 1998; Greenfield, 2009, 2016).

Interestingly, prior research has yielded mixed and even seemingly contradictory empirical findings. On the one hand, it is well-documented that some aspects of culture change such as the rise of individualism (Hamamura, 2012; Talhelm et al., 2014; Yu et al., 2016) as societies shift from traditional to urban, from poor to rich, from more isolated to more interconnected, from less educated to more educated, from more agricultural to more industrialized (Greenfield, 2016). For instance, as China has become wealthier, divorce rates, an indicator of modernity, have risen over the past decades (Talhelm et al., 2014). The same pattern between wealth and divorce seems to hold in societies around the world (Trent and South, 1989). On the other hand, accumulating evidence suggests that some societies such as Japan, Korea, and China remain highly collectivistic despite rapid economic growth and urbanization, posing a challenge to the modernization theory (Hamamura, 2012; Talhelm et al., 2014). For example, from 1990 to 2007, Japan, Korea, and China became much wealthier, yet agreement with “family is important in life” has been constant (Korea) or increasing (China and Japan; Talhelm, 2015). The World Values Survey has also shown that traditional or indigenous value systems are persisting in many societies even through modernization (Inglehart and Baker, 2000). Therefore, it is not clear how China’s recent modernization might be changing its culture and moral values.

As modernization and globalization broaden and deepen, we might think of Chinese people as no longer monocultural but rather bicultural or multicultural, having been greatly reshaped by a hybrid culture mixing traditional and modern cultures. This should be especially true for younger generations (e.g., people who were born and raised since the 1980s). Chinese people may construe bicultural or multicultural selves and develop multiple identities or personalities to mentally cope with this cultural change (Lu and Yang, 2006).

In response to large-scale societal modernization, social psychologists Yang (1998) proposed that Chinese people may develop two distinct kinds of psychological systems termed as “psychological traditionality” and “psychological modernity.” The concept of psychological traditionality is comprised of a set of concurrent traditional psychological characteristics or traits, such as filial piety and ancestral worship (Yang, 1996). The concept of psychological modernity is comprised of a set of concurrent modern psychological characteristics or traits, such as egalitarianism and open-mindedness (Yang, 1996). Yang (1996) argue that psychological traditionality and modernity co-exist in Chinese peoples’ mindsets and that each of them corresponds to relevant psychological processes and behavioral characteristics. Specifically, Yang (1996) found that a set of psychological characteristics has been dramatically reshaped by the modernization process in China. These included motivational, attitudinal, and temperamental changes (Yang, 1996). They also found that people who endorsed Chinese traditional culture emphasized familism, relationships, authority, and male dominance, whereas those who endorsed modern Chinese culture put more weight on achievements, autonomy, egalitarianism, utilitarianism, quality of life, and gender equality (Yang, 1996).

Although this line of work has offered us a solid theoretical framework and inspired much subsequent research (Leong and Chang, 2003; Pillutla et al., 2007), research to date has not yet closely examined whether psychological traditionality and modernity affect Chinese young adults’ moral concerns when two particular moral codes are in conflict with each other. That leaves important research questions unaddressed. For instance, do people who endorse Chinese traditional culture prioritize certain moral values (e.g., hierarchical structure, interpersonal obligations, and filial piety), while those who identify with Chinese modern culture prioritize other moral values (e.g., autonomy, justice, and liberty)? Are these core values derived from distinct cultural schemas compatible or incompatible with each other? How do people resolve moral dilemmas when being exposed to differing contextual cues?

Although cross-cultural perspectives on morality have been a flourishing research field in the past decades (for a review, see Haidt, 2007; Graham et al., 2016), some cultural psychologists have advocated for a paradigm shift from cross-cultural comparisons to a dynamic constructivist approach (Hong et al., 2000; Chiu and Hong, 2006; Hong, 2009). This view contends that culture and morality mutually shape each other and that morality is made of highly culturally conditioned responses rather than specific ties to particular individuals, groups, or communities. In other words, when being primed with specific cultural cues, even the same person with different identities will make different decisions depending on which identity is accessible and salient to them at the particular moment. This may be especially true when the participants are bicultural or multicultural persons such as immigrants or Hong Kong Chinese (Hong et al., 2000; Hong, 2009). Furthermore, past work with the cultural priming paradigm has demonstrated that subtle but powerful cultural cues can successfully shift people’s psychological processes and behavioral patterns (Hong et al., 2000; Hong and Chiu, 2001; Sui et al., 2007; Zou et al., 2008). It is thus interesting and plausible to examine the dynamic interplay of cultural change and moral concerns among Chinese young adults who are construed as traditional-modern bicultural individuals.

## An Overview of the Present Research

To date, theoretical formulations and empirical evidence on traditional/modern cultures and moral priorities in the context of Chinese cultural change have been sparse. This research adds further empirical evidence to the growing body of work on whether Chinese traditional and modern cultures relate to or influence young adults’ moral priorities.

Study 1 took an individual-difference approach and tested for the relations between psychological traditionality/modernity and moral priorities (justice vs. relationship). Study 2 took a dynamic constructivist approach and inferred casualty by using the cultural priming technique. Past work on cultural priming predominantly sampled either bicultural Chinese located in Hong Kong or bicultural immigrants who moved to North America or West Europe from their homelands (Hong et al., 2000). Our work instead targeted Chinese young adults who were born and raised in Mainland China after China launched the reform and opening policy in 1978. Our empirical logic is based on the assumption that these younger generations have been deeply influenced by a mix of ingrained Chinese traditional culture (e.g., through cultural transmission from older generations or implicit cultural norms) and foreign cultural inflow. These age cohorts or cultural groups have also experienced China’s large-scale modernization firsthand. It is also worth noting that this perspective echoes the conceptual distinction of globalization-based acculturation and immigration-based acculturation (Chen et al., 2016). People do not have to relocate to far-away lands to experience foreign cultural inflow, become bicultural individuals, and face the challenge of complex cultural adaptation. Many cultural priming studies show that when primed with distinct cultures, participants exhibit different attribution tendencies (Hong et al., 2000), self-referential effects (Sui et al., 2007), conflict-resolution strategies (Morris and Fu, 2000), and differing levels of interconnectedness (Ng and Lai, 2009). However, there is little direct data on whether Chinese cultural change affects people’s moral functioning such as thoughts, feelings, intentions, and behaviors. Drawing upon the premise that both justice and relationship concerns are accessible moral codes that are widely shared cultural knowledge for young Chinese, the cultural priming technique would thereby allow us to test for a causal link between Chinese traditional/modern cultures and moral priorities.

It is well-established that Chinese traditional cultures emphasize interpersonal obligations and particularistic role duties (Hwang, 1998). For instance, Hwang (1998) put forward a Confucian way of humanity: the principle of respecting the superior and the principle of favoring the intimacy. Based upon past work, we expect that psychological traditionality will positively predict relationship priority (H1). Conversely, as modernization and globalization broadens and deepens in Mainland China, younger generations may adopt the core values of WEIRD cultures (i.e., Western, educated, industrialized, rich, and democratic; Henrich et al., 2010), such as autonomy, justice, and liberty. They may advocate for right-based morality rather than duty-based morality (Chiu et al., 1997). We therefore anticipate that psychological modernity will positively predict justice priority (H2). The following two studies will directly test these predictions, respectively.

### Study 1: Are Psychological Traditionality and Modernity Associated With Moral Priorities?

Study 1 used a cross-sectional design to examine the link between psychological traditionality/modernity and moral priorities among Chinese young adults.

#### Pilot Study

For the purpose of our current study, we selected and modified 10 moral scenarios based on scenarios of moral dilemmas that have been widely used in past research (Kohlberg, 1981; Miller and Bersoff, 1992; Greene et al., 2001). We then conducted a pre-test in an independent sample (*N* = 60) to rule out a potential confound: the perceived importance of justice rule and relationship rule to exclude a possible ceiling effect or floor effect. Accordingly, we presented our participants cases of moral dilemmas to ensure they would perceive difficulty in resolving the moral dilemmas. We excluded four moral scenarios in which more than 90% of our participants prioritized justice concern over relationship concern or vice versa. The final set contains six moral scenarios (see the full details of our scenarios in Appendix A).

### Materials and Methods

#### Participants and Procedure

Participants were recruited via online advertisements in two universities in Beijing. Questionnaires were distributed during a class by research assistants and then returned to the researchers. A total of 221 college students participated in our study (142 females; age range = 18–35). Each of them was presented with a written informed consent document before filling out the questionnaire and was thanked for their participation.

#### Measures

##### Psychological traditionality

We used the 41-item psychological traditionality scale developed by Yang et al. (1991). It consists of five subdimensions: submission to authority, filial piety and ancestral worship, conservatism and endurance, fatalism and defensiveness, and male dominance (Yang, 1996). Endorsement of each item was assessed with a 7-point Likert scale (1 = *Strongly Disagree* and 7 = *Strongly Agree*). A sample item is “The best way to avoid mistakes is to obey what elders say.”

##### Psychological modernity

We used the 36-item psychological modernity scale developed by Yang et al. (1991). It consists of five subdimensions: egalitarianism and open-mindedness, social isolation and self-reliance, optimism and assertiveness, affective hedonism, and gender equality (Yang, 1996). Endorsement of each item was assessed with a 7-point Likert scale (1 = *Strongly Disagree* and 7 = *Strongly Agree*). A sample item is “If married life is too painful, divorce may be a way to solve the problem.”

##### Moral scenarios

Drawing upon classic work from Kohlberg (1981), Miller and Bersoff (1992), and Greene et al. (2001), we adapted a set of moral scenarios and asked participants to rate to what extent they would endorse the moral actor to make a moral decision based on the justice concern or the relationship concern on a 7-point Likert scale (1 = *Strongly Disagree* and 7 = *Strongly Agree*). For instance, we modified the classic trolley problem by creating a choice between saving a best friend’s life and saving five strangers’ lives. We asked participants the extent to which they would agree that the actor should pull the lever to save five people on the main track but in so doing kill his/her best friend on the side track. The other option was not to pull the level, letting the five strangers die, but saving his or her best friend’s life. The Cronbach’s alpha was 0.52 (see the six moral scenarios in Appendix B; results should be interpreted with caution due to relatively low internal consistency).

## RESULTS

Descriptive statistics and zero-order correlation matrix are displayed in Table 1. Using hierarchical multiple regression analyses, results indicated that psychological traditionality positively predicted relationship concern (*B* = 0.37, *SE* = 0.17, β = 0.15, *t* = 2.26, *p* = 0.02, Δ*R*^2^ = 0.02; H1 was supported; see Table 2) even after controlling for psychological modernity, gender, and age. Furthermore, psychological modernity was not related to justice concern (*B* = 0.16, *SE* = 0.18, β = 0.06, *t* = -0.85, *p* = 0.39, Δ*R*^2^ = 0.00; H2 was not supported; see Table 3) after parceling out psychological traditionality, gender, and age. These findings were consistent with the conceptual claims that individuals who score high in psychological traditionality would more likely endorse moral values such as collectivism, familism, and relationship orientation (Yang, 1996). Interestingly, however, individuals who scored high in psychological modernity did not report higher justice concern, suggesting that Chinese young adults may not mentally associate Chinese modern culture with justice concern. No gender or age effects were observed.

**Table 1:** Means, SD, and correlation matrix among the key measures.

	Mean	*SD*	1	2	3	4
1. Traditionality	3.86	0.57	-			
2. Modernity	5.31	0.52	0.08	-		
3. Relation	3.92	0.72	0.12	-0.14^∗^	-	
4. Justice	4.45	0.68	0.01	-0.04	-0.47^∗∗∗^	-


*N = 221; traditionality, psychological traditionality; modernity, psychological modernity; relation, relationship concern; justice, justice concern. ^∗^p < 0.05 and ^∗∗∗^p < 0.001.*

**Table 2:** Hierarchical multiple regression analyses predicting relationship concern from psychological traditionality.

Variables	Model 1			Model 2			Model 3		
	*B*	*SE*	β	*B*	*SE*	β	*B*	*SE*	β
Gender	-0.14	0.10	-0.10	-0.13	0.10	-0.09	-0.12	0.10	-0.08
Age	0.01	0.09	0.01	-0.01	0.09	0.01	0.02	0.09	-0.01
Modernity				-0.19	0.09	-0.13^∗^	-0.20	0.09	0.15^∗^
Traditionality							0.16	0.09	0.13^∗^
R2	0.01			0.03			0.04		
ΔR2				0.02			0.01		
F for ΔR2				3.92^∗^			3.61^∗^		


*N = 221; unstandardized (with SE) and standardized regression coefficients are displayed. Traditionality, psychological traditionality; modernity, psychological modernity. ^∗^p < 0.05, ^∗∗^ p < 0.01, and ^∗∗∗^p < 0.001.*

**Table 3:** Hierarchical multiple regression analyses predicting justice concern from psychological modernity.

Variables	Model 1			Model 2			Model 3		
	*B*	*SE*	β	*B*	*SE*	β	*B*	*SE*	β
Gender	0.14	0.10	0.10	0.14	0.10	0.10	0.14	0.10	0.10
Age	0.07	0.08	0.06	0.07	0.08	0.06	0.06	0.08	0.02
Traditionality				0.02	0.08	0.02	0.03	0.08	0.02
Modernity							-0.06	0.09	-0.04
R2	0.01			0.01			0.01		
ΔR2				0.00			0.00		
F for ΔR2				0.07^∗^			0.41		


*N = 221; unstandardized (with SE) and standardized regression coefficients are displayed. Traditionality, psychological traditionality; modernity, psychological modernity. ^∗^p < 0.05.*

### Study 2: Can Priming Chinese Traditional or Modern Cultures Affect Moral Priorities?

Study 2 adopted the cultural priming paradigm to infer the causal relations between Chinese traditional/modern cultures and moral priorities among Chinese young adults.

#### Pilot Study

Given that most of the cultural icons used in prior cultural priming studies reflect national cultures (e.g., American vs. Chinese flag), they do not serve the purpose of the current research. To select cultural symbols well suited to this study, we first invited an independent group consisting of 80 participants (50 females; age range = 18–35) to generate the most culturally representative objects or figures from a selection of more than 600 cultural objects (e.g., architecture and historical figures). They identified 40 pictures representing Chinese traditional culture and another 40 pictures representing Chinese modern culture. According to the level of cultural representativeness, we selected the final set of cultural icons including 16 images of Chinese traditional culture (e.g., Confucius and the Great Wall), 16 images of Chinese modern culture (e.g., Deng Xiaoping and the modern Shanghai), and 16 natural landscape pictures for the control group (See all the cultural icons in Appendix B).

### Materials and Methods

#### Participants

A total of 73 Chinese college students in two public universities located in Beijing participated in our experiment (55 female; *M*age = 21.53, *SD*age = 2.06). The whole experimental session lasted about 30 minutes. Each participant was presented with a written informed consent, compensated with 20 RMB, debriefed and thanked for their participation.

#### Materials and Procedure

After coming to the lab, participants were told that the experiment consisted of two independent sessions to examine their pictorial cognition and social judgments. In session one, participants were randomly assigned to one of three conditions: traditional Chinese culture, modern Chinese culture, and neutral condition. Each of the 16 cultural icons for each priming condition was presented for 5 seconds on a slide show (Liu et al., 2017), with no time gap between pictures. Participants were then asked to write three sentences to describe the 16 images, which we added as a manipulation check. In the neutral condition, participants viewed 16 pictures of natural landscapes and were asked to write three sentences about how they had spent their day. In session two, participants read the six moral scenarios and indicated their moral priority. To gauge our participants’ baseline attitudes, they were invited back 3 weeks later to measure their general tendency without any priming. The materials were identical to those used in Time 1 (Cronbach’s alpha was 0.45 for Time 2).

## RESULTS

Using a 3 (cultural priming conditions) × 2 (repeated measure for moral scenarios) two-way ANCOVA, with gender and age as covariates, results indicated that participants did not prioritize relationship or justice concern when primed with traditional culture or modern culture or control stimuli, *F(2, 70) = 0.56, p* = 0.57, Δ*η*^2^ = 0.02. No cross-temporal difference in moral priorities was observed, *F(1, 71) = 1.27, p* = 0.26, Δ*η*^2^ = 0.02. Moreover, the interaction effect was also non-significant *F(2, 4970) = 0.29, p* = 0.75, Δ*η*^2^ = 0.01. These null results on the link between modernity and justice are consistent with the lack of empirical support for the modernization hypothesis. Alternatively, our participants’ moral priorities may be quite stable psychological tendencies, which are refractory to being influenced by temporarily priming of subtle contextual cues.

## General Discussion

Two studies tested the relations between Chinese traditional/modern cultures and moral priorities among bicultural young Chinese. Together, our findings show that psychological traditionality was related to higher priority of relationship concern in moral dilemmas. This result indicates that young Chinese still mentally associate traditional culture with moral value of interpersonal obligations despite large-scale modernization. Since traditional Chinese culture is heavily influenced by Confucianism philosophies that emphasize particularistic role obligations, participants who strongly endorse traditional culture feel morally obligated to prioritize interpersonal expectations.

However, neither individual differences in endorsements of modern culture nor priming modern icons were linked to people’s endorsement of justice concern in moral dilemmas. These insignificant results suggest that modernization plays out differently across different cultures, distinguishing modernization from individualism. One possible interpretation is that pervasive modernization does not necessarily lead Chinese people to endorse or internalize justice principles, as modernization theory predicts. An alternative explanation may be that Chinese modern culture which our 16 cultural icons represent are deeply woven into participants’ everyday life and become a natural part of living (Oyserman et al., 2002); priming modern cues thus may not successfully activate their corresponding moral code such as justice concern. Future research should directly and empirically test these possibilities.

### Contributions and Implications

Our studies yield some contributions and implications for theory and research on morality and culture. First, our data revealed interesting patterns on the relations between traditional/modern Chinese culture and moral priorities, adding to the growing body of work on how differing (sub)-cultures influence moral judgments. Our findings also provide direct evidence to support the sociological claim that traditional value systems persist despite the pervasive process of globalization and modernization in China (Inglehart and Baker, 2000). One important distinction between previous work and our research is that past studies mostly focus on bicultural individuals such as Chinese Americans as a result of immigration or Hong Kong Chinese as a result of colonization who endorse or internalize two cultural systems. Our studies instead examined bicultural young Chinese who have been immersed in traditional and modern Chinese cultures because of rapid and deep cultural change since 1978. Practically speaking, mapping out the dynamic interplay of Chinese traditional/modern culture and moral values can shed new light on uncovering the moral puzzle in modern China. Advancing our understanding of the upsides and downsides of cultural change in the moral domain is also vital to minimize moral losses while reaping the benefits of modernization.

### Limitations and Future Directions

Our work also contains some limitations. First, the measures of moral priorities are self-report and the internal consistency was not high. Thus, results are not conclusive and are also susceptible to social desirability and reference-group effects (Heine et al., 2002). Second, our current data were obtained from college students, which limit our ability to make boarder generalizations. Nonetheless, our findings demonstrate interesting patterns of results and open up new avenues for future research. Future work can obtain more robust and compelling evidence by further examining the effects of traditional/modern Chinese cultures on individuals’ moral thoughts, feelings, and behaviors. It would also be interesting to empirically test whether self-construal plays a mediating role (Markus and Kitayama, 1991). Future work should also go beyond samples of college students by including diverse age cohorts, rural populations, ethnic minorities, religious groups, or individuals who possess rich multicultural experiences. It is our hope that our work will inspire more high-quality work to uncover the dynamic interplay between culture and morality especially within the context of profound cultural change in mainland China.

## Ethics Statement

This study was approved from the Institutional Review Board for the Protection of Human Subjects at Beijing Normal University.

## Author Contributions

XH conceived the core research idea, designed the experiment, collected and analyzed the data, and drafted the manuscript. KP and LL supervised the project and edited the manuscript. SC, LZ, and FY provided comments, suggestions and critical revisions.

## Conflict of Interest Statement

The authors declare that the research was conducted in the absence of any commercial or financial relationships that could be construed as a potential conflict of interest.
