# Supplementary material for: Do Chinese Traditional and Modern Cultures Affect Young Adults’ Moral Priorities?
Source: Front Psychol. 2018 Nov 6;9:1799. doi: 10.3389/fpsyg.2018.01799 (PMC6232185; doi:10.3389/fpsyg.2018.01799)
Supplement: Supplementary file 1 [file Image_1.pdf]

Appendix A: The Moral Scenarios

1. One night, Li Rui is driving and Zhao Gang is with him. Suddenly, they hit a pedestrian because Li is speeding. The pedestrian is seriously hurt, and Li is sued to the court for a large fine and possible imprisonment. There is no witness except for Zhao. Li's lawyer tells Zhao that if he could testify that Li did not speed, Li may avoid imprisonment. Zhao and Li grew up together, were classmates in primary and secondary school, and are still best friends. If Zhao testifies, Li could avoid imprisonment, but the pedestrian will not be properly compensated. Zhao is facing a hard choice.

To what extent would you agree with the following statement?

A. Zhao should not testify, because he has a duty to respect the rights of the pedestrian.

[1]	[2]	[3]	[4]	[5]	[6]	[7]
Strongly disagree	Moderately disagree	Slightly disagree	Neutral	Slightly agree	Moderately agree	Strongly agree

B. Zhao should testify, because he has a duty to help Li avoid imprisonment.

[1]	[2]	[3]	[4]	[5]	[6]	[7]
Strongly disagree	Moderately disagree	Slightly disagree	Neutral	Slightly agree	Moderately agree	Strongly agree

2. Liu Tao is a train driver. One day, when driving the train, he finds that there is a worker on the tract, and that worker happens to be his best friend Li Shan. Li has no chance to escape, so Liu hit the brake, but it does not work. He realizes that he can turn the train to another track to save his friend's life, but doing so will hit 5 other workers on that track. Liu and Li have been best friends for decades, and Liu really wants to save him, but if he turns the train, he would hit 5 other workers. Liu is now in a dilemma.

To what extent would you agree with the following statement?

A. Liu should turn the train, because he has a duty to protect his friend's life.

[1]	[2]	[3]	[4]	[5]	[6]	[7]
Strongly disagree	Moderately disagree	Slightly disagree	Neutral	Slightly agree	Moderately agree	Strongly agree

B. Liu should not turn the train, because he has a duty to protect those 5 workers.

[1]	[2]	[3]	[4]	[5]	[6]	[7]
Strongly disagree	Moderately disagree	Slightly disagree	Neutral	Slightly agree	Moderately agree	Strongly agree

3. Wang Chao is having a trip to Shanghai due to work requirements. After that, he hurries to the train station for a trip to Nanjing. He must attend Zhou Ping’s wedding, as the witness. Suddenly, he realizes that his purse was stolen, in which were all his money and his train ticket. He asked for help, but no one can give any. Then he sees a man put a coat on the seat next to him. He notices that a tick drops from the pocket, and that ticket happens to be one for the next train to Nanjing. He must catch this train to get to Zhou’s wedding in time. Wang and Zhou are best friends in college, so Wang thinks he must be in Zhou’s wedding, but taking other people’s ticket obviously violates their interest.

To what extent would you agree with the following statement?

A. Wang should not take the ticket, because he has a duty not to harm other people’s interest.

[1]	[2]	[3]	[4]	[5]	[6]	[7]
Strongly disagree	Moderately disagree	Slightly disagree	Neutral	Slightly agree	Moderately agree	Strongly agree

B. Wang should take the ticket, because he has a duty to make it to his best friend’s wedding.

[1]	[2]	[3]	[4]	[5]	[6]	[7]
Strongly disagree	Moderately disagree	Slightly disagree	Neutral	Slightly ee	Moderately agree	Strongly agree

4. Lu Yu has a seizure and doctor says she must take surgery as soon as possible, otherwise he may be forever paralyzed. The surgery takes a fortune. Lu's family tries to borrow money from everyone they knew, but has only collected half the fee. Zhang Yue is a manager of an investment fund, and best friend of Lu. They are very close since high school and remain so all the way through college. Zhang really wants to help her friend and tried everything she can, but the number is too high. She suddenly realizes that she could her client's fund and then try to make it up later. However, doing so will seriously hurt her client's interest, causing large amount of loss.

To what extent would you agree with the following statement?

A. Zhang should not use that fund, because she has a duty not to put her client in jeopardy.

[1]	[2]	[3]	[4]	[5]	[6]	[7]
Strongly disagree	Moderately disagree	Slightly disagree	Neutral	Slightly agree	Moderately agree	Strongly agree

B. Zhang should use that fund, because she has a duty to help her best friend.

[1]	[2]	[3]	[4]	[5]	[6]	[7]
Strongly disagree	Moderately disagree	Slightly disagree	Neutral	Slightly agree	Moderately agree	Strongly agree

5. Zhao Di is in charge of recruitment in her company. One applicant named Deng Hong is a perfect fit for the position. Yuan Lei has also applied for this position. She is Zhao's best friend and helps her whenever she is in need. Yuan is a little behind Deng for this job. Yuan has asked Zhao to give her this position several times. Zhao really wants to give it to Yuan, but doing so will ruin Deng's opportunity, and will violate the company's regulations.

To what extent would you agree with the following statement?

A. Zhao should choose Yuan, because she has a duty to help her friend get the job.

[1]	[2]	[3]	[4]	[5]	[6]	[7]
Strongly disagree	Moderately disagree	Slightly disagree	Neutral	Slightly agree	Moderately agree	Strongly agree

B. Zhao should not choose Yuan, because she has a duty to follow the company's regulations.

[1]	[2]	[3]	[4]	[5]	[6]	[7]
Strongly disagree	Moderately disagree	Slightly disagree	Neutral	Slightly agree	Moderately agree	Strongly agree

6. Yang Jia is a surgeon in charge of kidney transplant in a large hospital. Due to the lack of donator, patients often have to wait for half a year, or even longer. One day, a new kidney arrives and Yang is going to inform the next patient on the waiting list, a man named Wei Hua. Suddenly, Yang's best friend Zhen Xin comes to him, saying that he was just diagnosed with kidney failure and needs transplant as soon as possible. The new kidney happens to match Zhen's requirement. Zhen and Yang have been best friends for decades, and they treat each other as families. Yang now faces a hard choice, as either Wei or Zhen could lose his life if he does not receive transplant.

To what extent would you agree with the following statement?

A. Yang should help Zhen, because he has a duty to help his best friend.

[1]	[2]	[3]	[4]	[5]	[6]	[7]
Strongly disagree	Moderately disagree	Slightly disagree	Neutral	Slightly agree	Moderately agree	Strongly agree

B. Yang should not help Zhen, because he has a duty for the patients on the waiting list.

[1]	[2]	[3]	[4]	[5]	[6]	[7]
Strongly disagree	Moderately disagree	Slightly disagree	Neutral	Slightly agree	Moderately agree	Strongly agree
