# Supplementary material for: Do Chinese Traditional and Modern Cultures Affect Young Adults’ Moral Priorities?
Source: Front Psychol. 2018 Nov 6;9:1799. doi: 10.3389/fpsyg.2018.01799 (PMC6232185; doi:10.3389/fpsyg.2018.01799)

Appendix B: The Cultural Icons

1. Pictorial Icons for the Chinese traditional culture condition

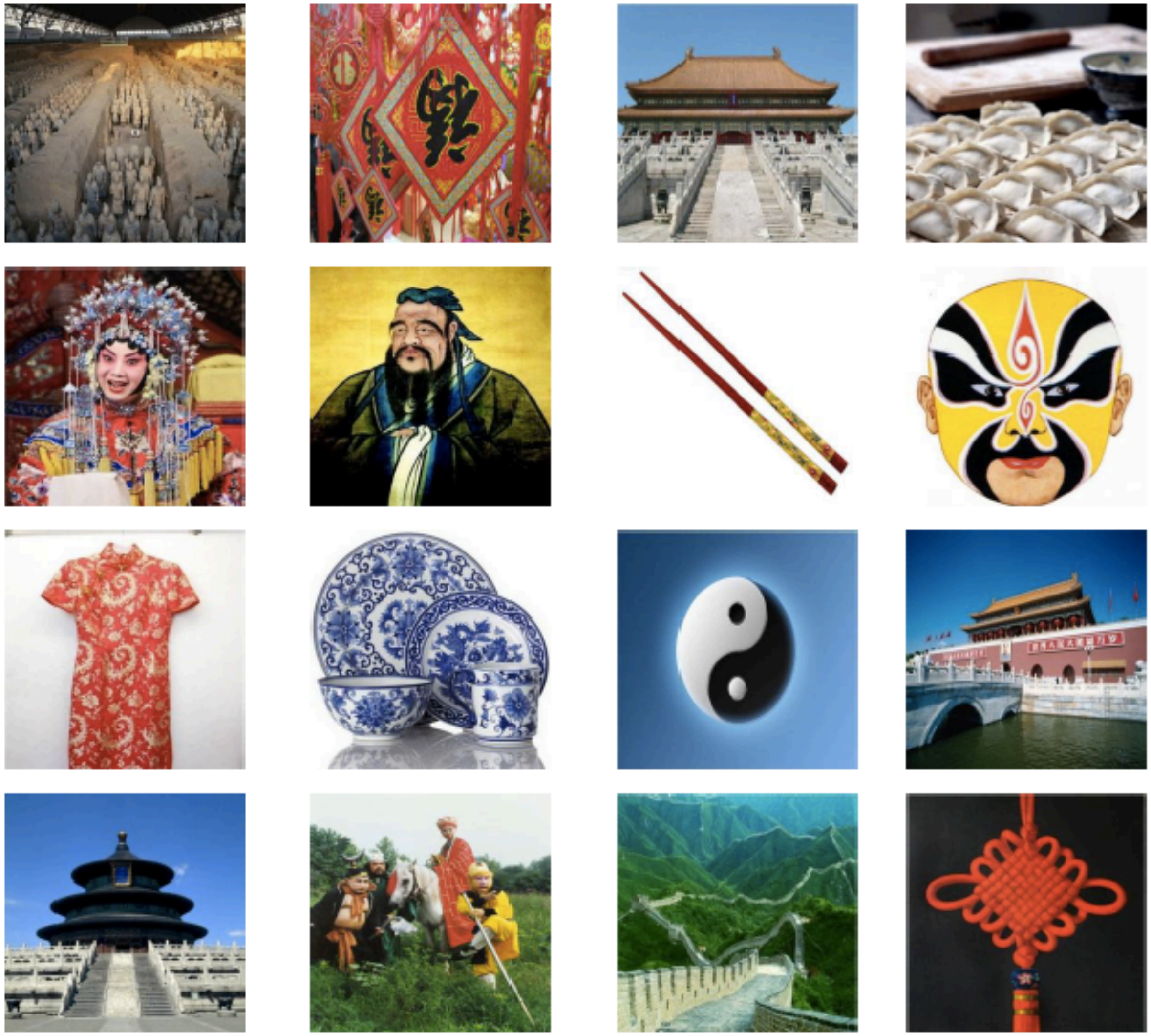

2. Pictorial Icons for the Chinese modern culture condition

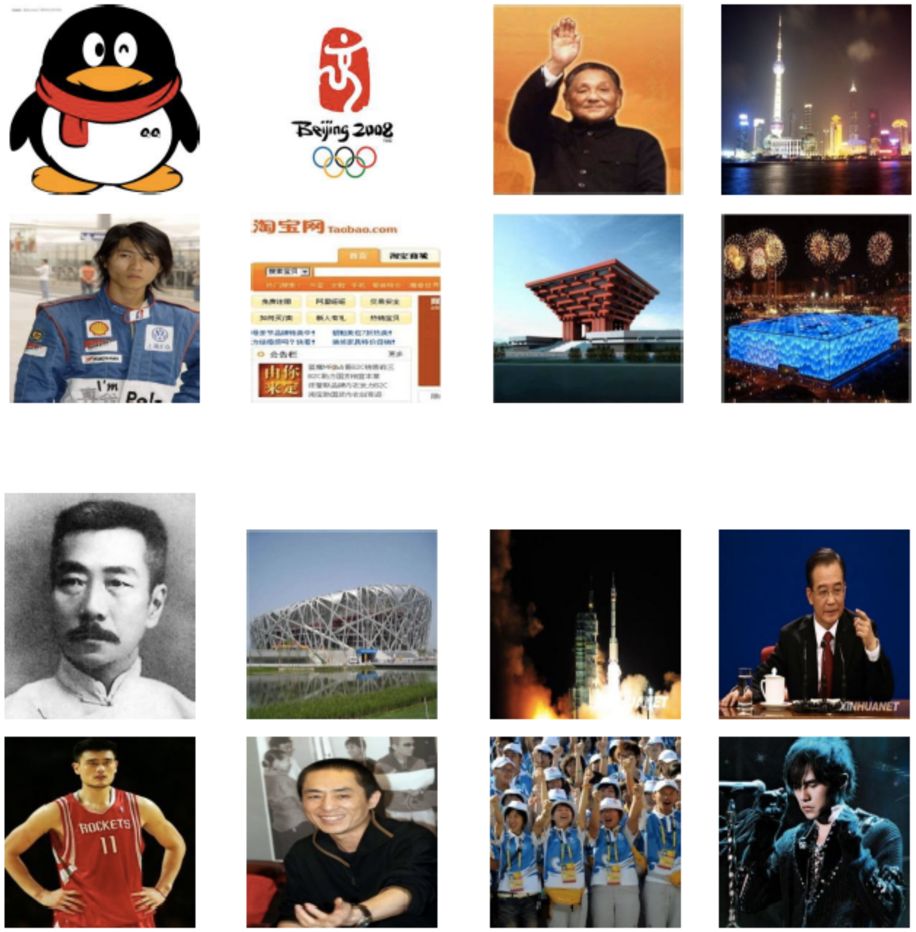

3. Pictorial Icons for the culturally neutral condition

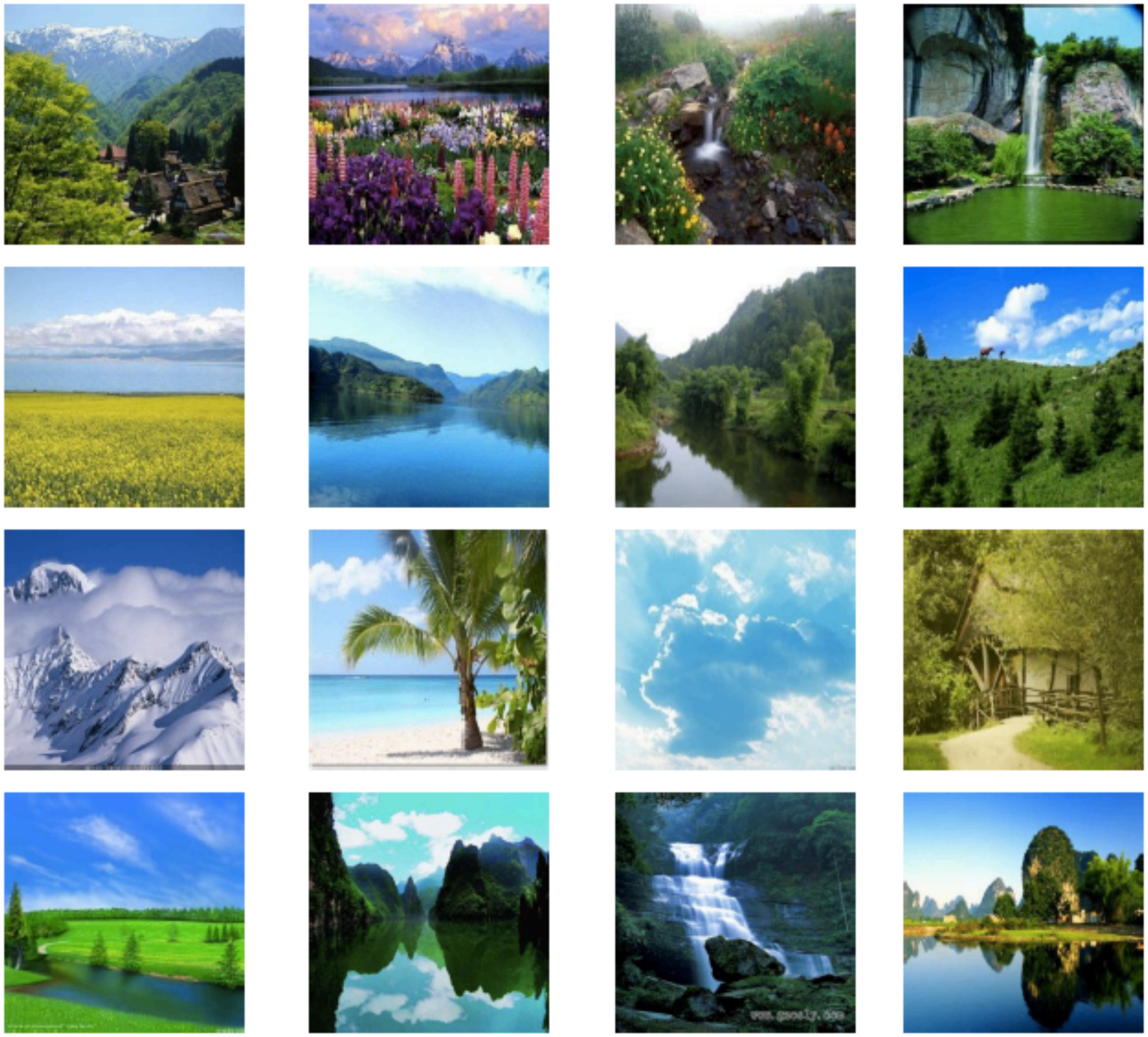

Supplement: Supplementary file 2 [file Image_2.pdf]
